# Mycotoxin-Linked Mutations and Cancer Risk: A Global Health Issue

**DOI:** 10.3390/ijerph19137754

**Published:** 2022-06-24

**Authors:** Theodora Ekwomadu, Mulunda Mwanza, Alfred Musekiwa

**Affiliations:** 1 Food Security and Safety Niche Area, Department of Animal Health, Mafikeng Campus, Northwest University, Mmabatho 2735, South Africa; mulunda.mwanza@nwu.ac.za; 2 Division of African Cancer Institute, Department of Global Health, Faculty of Medicine and Health Sciences, Stellenbosch University & Tygerberg Hospital, Cape Town 7505, South Africa; 3 Division of Epidemiology and Biostatistics, Department of Global Health, Faculty of Medicine and Health Sciences, Stellenbosch University & Tygerberg Hospital, Cape Town 7505, South Africa; alfred.musekiwa@gmail.com

**Keywords:** aflatoxin, fumonisin, cancer, mutation, fungal metabolites, food, exposure, mycotoxins

## Abstract

Humans continue to be constantly exposed to mycotoxins, mainly through oral exposure (dietary), inhalation, or dermal contact. Recently, it has been of increasing interest to investigate mycotoxin-linked carcinogenicity. This systematic review was conducted to synthesize evidence of the association between mycotoxin-linked mutations and the risk of cancer, to provide an overview of the data linking exposure to different mycotoxins with human cancer risk, and to provide an update on current research on the risk of cancer associated with human exposure to mycotoxins. PRISMA guidelines were used when conducting the systematic review. PubMed, MEDLINE, and CINAHL electronic databases were comprehensively searched to extract the relevant studies published from inception to May 2022. A total of sixteen relevant studies (4907 participants) were identified and included in this review. Of these, twelve studies were from Asia, while four of the studies were conducted in Africa. The overall meta-analysis result found no significant association, although some of the studies confirmed an association between mycotoxin-linked mutations and primary liver cancer risk. Mainly, the experimental studies have shown associations between mycotoxin-linked mutations and cancer risk, and there is a need for researchers to confirm these links in epidemiological studies in order to guide public health policies and interventions.

## 1. Introduction

Mycotoxins are toxic fungal metabolites which are structurally diverse and cause adverse human and animal health issues, mainly by oral exposure. They are the common contaminants of agricultural food/feed products, such as maize, wheat, nuts, and other foods derived from them. To date, mycotoxins with carcinogenic potency as reported in the literature include aflatoxins, ochratoxin, fumonisins, zearalenone, and some Penicillium toxins. Most of these carcinogenic mycotoxins are genotoxic and mutagenic agents in many model systems and produce chromosomal aberrations. In various climatic conditions, fungi can produce different mycotoxins, while some mycotoxins can be produced by different fungal species [1]. However, this can cause coexposure to many mycotoxins, with the resultant harmful health effects which includes cancer risks. Types of mycotoxins, and how often one is exposed to them, can affect disease expression, and possibly have synergistic effects with other chemical compounds to which the person is exposed [2]. Furthermore, mycotoxins can exert either acute or chronic toxicities through rapid onset or slow progression over an extended time period of low-dose mycotoxin exposure. This invariably may lead to malignant tumours and adverse health effects [2]. Mycotoxins can be absorbed from contact sites such as the GIT or respiratory tract and reach the vital organs, where the toxin distributes all over the body [3]. Mycotoxins can penetrate human as well as animal cells, causing permanent damage and mutations. During the normal processes of cell division, those mutations can show-up and potentially aggravate normal cellular growth [3]. A number of cell processes, for example protein synthesis, are affected by some mycotoxins such as ochratoxin A (OTA) and deoxynivalenol (DON). In addition, aflatoxin B1 was shown to have carcinogenic effects and also cause DNA damage by the IARC [2]. As a result of coexposures to various mycotoxins, the occurrence of complex interactions in the cells has been suggested which could possibly result in synergistic impacts [2]. This review therefore attempts to briefly summarize the currently available data on mycotoxin-linked mutations caused by the major mycotoxins and their links and or roles in human cancer development. To our knowledge, there is no current or ongoing systematic review on this topic and only a few human epidemiological studies have investigated the link between mycotoxin exposure and cancer risk [4].

### 1.1. Factors Influencing Fungal and Mycotoxin Contamination of Agricultural Commodities

A variety of factors influence the fungal and mycotoxin contamination of agricultural commodities. Among them are physical/environmental, chemical, and biological factors [5]. Physical/environmental factors include time, the extent of insect damage, abiotic factors such as physiographic factors (location and topography), climatic factors (temperature, light, air pressure and wind, rainfall, and humidity), edaphic factor/soil composition (such as clay, loam or sand, the pH of the soil, mineral salts and trace elements, and water-holding capacity), and gases (vapour, oxygen, carbon dioxide, and nitrogen) [6,7]. Chemical factors include fertilizers, fungicides, and other chemicals which are usually used in farming. It has been shown that these chemicals might encourage the production of mycotoxins. The successful control of diseases can be achieved using fungicides but not to control mycotoxin production. Some studies have found that sublethal concentrations of some of these chemicals can proliferate mycotoxin production [8]. The effect of fungicides on mycotoxin production are not consistent and are influenced by other environmental factors [8]. In addition, nitrogen and carbon sources, with trace metals, often present in fertilizers, even have an effect on mycotoxin production [9,10,11]. For instance, an increase in the nitrogen concentration in fertilizers can cause an increase in DON production by *F. graminearum* [12].

Lastly, one of the biological factors influencing mycotoxin production is the interaction between toxigenic fungal species and the substrate. Among the factors are strain specificity, strain variation, and eventually the instability of toxigenic properties [13,14]. However, mycotoxins can penetrate human cells, as well as animal cells, causing permanent damage and mutations. During the normal processes of cell division, those mutations can show-up and potentially aggravate normal cell growth [3].

### 1.2. Mycotoxins and Human Health

Mycotoxins with carcinogenic potency as reported in the literature include aflatoxins, fumonisins, ochratoxin, T2, zearalenone, and some *Penicillium* toxins [3].

Aflatoxin (AF): Aflatoxin B1 is the most carcinogenic among all mycotoxins, with the liver as its predominant target [15]. In humans, the occurrence of aflatoxin B1 and its metabolites have been reported in some organs such as the kidney, the heart, as well as in brain tissues, urine, or faeces. It is able to enter the cell membrane and fasten to its DNA, altering the genome so that it becomes more stable [16,17]. After it penetrates the cell membrane, it alters the cell cycle and affects the P53 gene. The P53 gene is responsible for encoding the tumour suppressor protein which obstructs the growth of tumours and cancers [18,19]. Moreover, aflatoxins were linked to many human diseases, such as Reyes syndrome, cancer of the liver, chronic gastritis, aflatoxic hepatitis, kwashiorkor, etc.

According to Peraica et al., [20] fumonisins were linked to oesophageal cancer in South Africa, China, and Northeast Italy. Fumonisin B1 was also linked to neural tube defects in babies whose mothers consumed maize contaminated with fumonisin along the Mexico boundary [21]. It has been shown that fumonisin B1 is associated with renal carcinomas of male rats, and also with liver cancer in female rats, which could also occur in humans [22]. Furthermore, fumonisin B1 obstructs ceramide synthase production in vitro and enhances tumour necrosis factor α production, which initiates apoptosis [23].

Ochratoxin A (OTA): Among the *Aspergillus* toxins, only OTA is potentially as important as the aflatoxin [24]. The kidney is the primary target organ, and it has been speculated that OTA is associated with Balkan endemic nephropathy in humans [24]. It is also suspected of contributing to chronic interstitial nephropathy in North Africa. Ochratoxin A and aflatoxins were also detected in the urine samples of children in Sierra Leone [25]. Furthermore, it was also hypothesized that OTA could be a risk factor in the aetiology of testicular cancer [24]. However, while ochratoxin A interrupts the physiology of the cell in many ways, it primarily affects the enzymes responsible for phenylalanine metabolism, which inhibits phenylalanine-tRNA complex synthesis [3,26]. Additionally, ochratoxin A can inhibit the production of mitochondrial ATP and is also known as a strong stimulant during lipid peroxidation [27].

T-2 toxin is one of the *Fusarium* mycotoxins, which usually contaminate unharvested grains that are left for a long time in the field. T-2 toxin is also caused alimentary toxic aleukia (ATA), which led to the death of many people in Russia. Alimentary toxic aleukia (ATA) is characterized by fever, skin, and nose bleeds, necrosis, and, apart from its cytotoxicity, also suppresses the immune systems of the affected individuals [24]. T-2 toxin also causes the DNA of lymphocytes to have breaks when administered in vivo. The DNA breaks also occur in vitro when the fibroblast cells are treated with T-2 toxin with 3 h thymidine added [3].

Zearalenone (ZEA) is also a *Fusarium* mycotoxin. It is a common contaminant of maize but can also affect other crops [28]. Being an endocrine disruptor, ZEA is assumed to decrease male fertility in both human and animal populations. It was also claimed that the high frequency of early menarche in Puerto Rico may be due to consuming ZEA-contaminated maize and related compounds in the diet [24]. Due to its ability to cause changes in the reproductive organs and systems of many laboratory animals, it was hypothesized that zearalenone can cause reproductive organ cancer in both humans and animals. Zearalenone was also shown to cause cancer in rats, e.g., hepatocellular and pituitary tumours [29]. Therefore, there is a need to further confirm its cancer-causing potential in humans. Regardless of it being toxic and carcinogenic, zearalenone is used in some countries for cattle production, as it increases meat production, while it is banned in some other countries [30], perhaps because there is not enough evidence/data that supports it being toxic or carcinogenic to humans and animals [31].

## 2. Materials and Methods

The Preferred Reporting Items for Systematic Reviews and Meta-Analyses guidelines (PRISMA) [32,33] were followed in this systematic review.

### 2.1. Literature Search

This systematic review considered all original studies done on human exposure to different mycotoxins and the associated cancer risk. Both cohort and case control study designs were eligible. The databases searched included: PubMed/MEDLINE, CINAHL, grey literature through Google Scholar, and the reference lists of the papers reviewed. The main Boolean operators (AND, OR) were used to combine search terms in different combinations. Search terms included combinations of: (mycotoxins), (fungal metabolites), (aflatoxins), (ochratoxin A), (fumonisins), (deoxynivalenol), (neoplasms), (mutations), (cancer), (exposure), (human). The search included articles published from inception up to 31 May 2022, and only articles published in the English language were searched.

### 2.2. Inclusion Criteria

Eligibility criteria for inclusion in the review were as follows: (1) Studies that investigate the link between any mycotoxin-linked mutations and risks of one or more types of human cancer; (2) Case-control and cohort study designs; (3) Relative risk (RR) or odds ratio (OR) estimates with 95% confidence intervals (CIs) reported, or data to calculate them.

### 2.3. Exclusion Criteria

Studies were excluded if they were animal studies, drug trials, diagnostic trials, case reports, studies only reporting qualitative findings, interventional studies, or studies not focusing on the association between mycotoxin-linked mutations and risk of one or more type of cancers in humans.

### 2.4. Data Extraction and Management

Data extraction and management were done independently and in duplicate by two review authors (TI and AM). Titles and abstracts retrieved from various electronic bibliographic databases were first screened for inclusion, and then full texts were examined in detail and screened for eligibility. Reference lists of eligible studies were hand-searched for additional articles. Data were extracted from articles using a template designed for this review. The data extracted from each study included: The first author’s last name, publication year, study population, study designs and sample size, follow-up period, gender, age, number of cases, exposure, outcome measurement, outcome measurement method, biomarkers, outcomes of significance to the review question, and objectives. Odds ratios (OR) and risk ratios (RR), with their corresponding 95% confidence intervals (CIs) reported, and variables adjusted for each measurement outcome in the analysis or data to calculate these if not already calculated, were also extracted.

### 2.5. Assessment of Methodological Quality of Included Studies and Data Synthesis

The quality and risk of bias of the selected studies was assessed independently and in duplicate by two review authors (TE and AM) using a modified Newcastle–Ottawa Scale for observational studies [34] and they were evaluated for methodological validity and the bias of epidemiological studies prior to inclusion in the review. Each study was evaluated using predefined criteria, and stars were awarded based on the selection of the study groups and the representability of the studied population, the comparability of the groups, and the exposure or the outcome of interest. Low scores indicate low quality and high risk of bias.

Where results were reported in a similar manner, and where there were no significant differences in results, for instance in the case of primary liver cancer (PLC), odds ratios were pooled using a random effects meta-analysis carried out in Stata (version 16, TX, USA). Heterogeneity between results in the meta-analysis was determined using both the Chi-square test (with *p*-value < 0.1 indicating significance) and I-square statistic (≥ 50% indicated substantial heterogeneity). Meta-analysis results were displayed using a forest plot.

## 3. Results

### 3.1. Results of the Search

**Figure 1:**
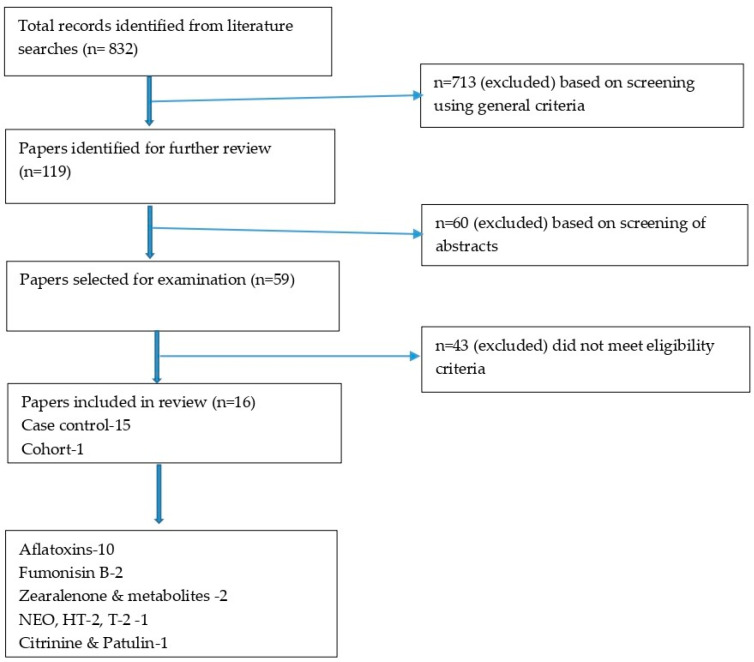
PRISMA chart for studies on mycotoxin-linked mutations and cancer risk.

The step-by-step process of the literature search and selection procedure is summarized in Figure 1. A total of 832 studies were identified through the systematic search. After excluding duplicates, the remaining 119 studies were screened, and 59 studies were then retained for full-text review. Finally, this systematic review incorporated 16 relevant studies, of which 11 studies were included in a meta-analysis.

### 3.2. Characteristics of the Included Studies

The sixteen included studies comprised of fifteen case–control studies [35,36,37,38,39,40,41,42,43,44,45,46,47,48] and one cohort study [49]. Twelve (75%) of the studies were from Asia [35,36,37,38,40,42,43,44,45,48,49], while the remaining four (25%) of the studies were conducted in Africa. The studies from Africa were carried out in South African [41], Sudanese [39], and, in two studies, from Tunisian populations [46,47]. The sixteen included studies were published between 1982 and 2021, with nine of them published between 2000 and 2021. Most of the studies provided adjusted risk estimates (OR, RR). Ten articles investigated the link between aflatoxin and primary liver cancer. Primary liver cancer includes hepatocellular carcinoma (HCC), intrahepatic cholangiocarcinoma (ICC), and some other extremely rare ones. Among the ten studies, seven reported on hepatocellular carcinoma (HCC), one study reported on cholangiocarcinoma, while two studies did not state which type of primary liver cancer. Besides aflatoxin, two articles also investigated the association between fumonisin B1 exposure and primary liver cancer (PLC) of the HCC type. Furthermore, two articles investigated zearalenone, (ZEA) and its cancer-causing potential on cervical and breast cancer. Aflatoxins were the most commonly studied mycotoxins, followed by fumonisin B1 and then ZEA, DON, HT-2, T-2, and C. None of the articles studied the health effects of some mycotoxins, such as OTA, or even the emerging mycotoxins. Different exposure matrices were being studied; for instance, seven of the articles studied blood and serum/plasma, seven articles used urine, then two articles food, and another two articles used toenails as their matrices of exposure assessment. Moreover, some exposure matrices studied included liver tissue, dust, and faeces, which had one article each. The studies have a wide variation in sample sizes. Two studies got as few as 27 and 58 participants [39,48], while the largest number of participants was on a study on liver cancer with 1102 participants [43]. A summary of the study characteristics and the findings of the included studies is provided in Table 1.

### 3.3. Results on the Quality of Studies Using the Modified Newcastle-Ottawa Scale (NOS) for Observational Studies

The study quality was evaluated using the Newcastle–Ottawa Scale, and the results are summarized in Table 2. This tool uses predefined criteria and awards stars for each article: four for quality of participant selection, two for comparability between cases and controls, and three stars for the adequate ascertainment of exposure, which in this study was the ascertainment of cancer risk. Hence, providing a total of nine achievable stars. Generally, the articles scored between 0 and 7 stars. A few studies had no proper study design, and some gave no data on the participants. Mostly, the studies had good scores on the selection category: five studies had four stars, six studies received three stars, two studies had two stars, and one study had one star. Two studies scored zero for selection, mostly because the selection of controls was not appropriate. In the comparability category, ten studies received two stars each, whereas five studies had one star each. The exposure category had most of the studies scoring only one star, with one study receiving three stars and one receiving two stars. Most of the scores were low and caused by not having ascertainment of the exposure.

### 3.4. Results of the Association on Mycotoxin-Linked Mutations and Cancer Risk

**Figure 2:**
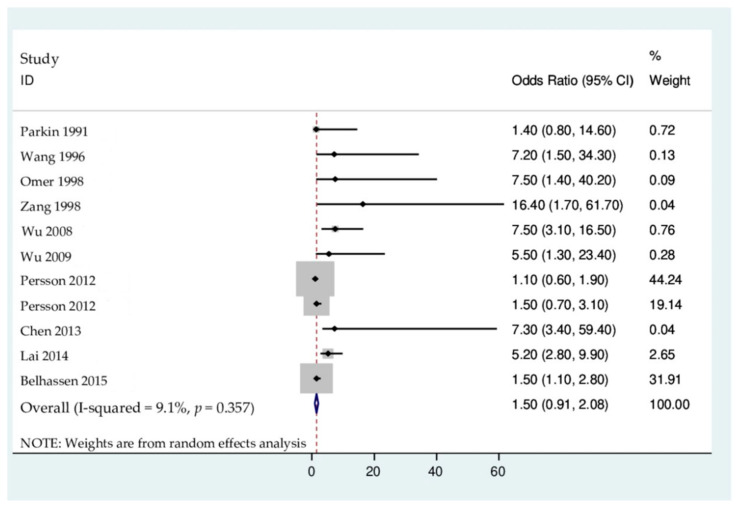
Forest plot of pooled meta-analysis result on association between mycotoxin-linked mutations and cancer risks [36,38,39,40,42,43,44,45,46,49].

Results are summarized in Table 3. Eleven studies assessed the association between aflatoxins and PLC and reported results in the form of odds ratios (OR) with corresponding 95% CIs. Individual results ranged from very little association (OR 1.40, 95% CI: 0.80, 14.6) in Parkin et al. [36] to a strong association (OR 16.4, 95% CI: 1.70, 61.70) in Zang et al. [40]. The results were pooled in a meta-analysis, which resulted in an overall meta-analysis showing no significant association (OR 1.50, 95% CI: 0.91, 2.08), and there was no significant heterogeneity between studies (Chi-square = 11.00 (degrees of freedom (df) = 10), *p* = 0.357, I^2^ = 9.1%) (Figure 2). For breast cancer, Belhassen et al. [46] found a significant association between the mycotoxin α-zearalanol and breast cancer (OR 1.54, 95% CI: 1.10, 2.77). The other study on breast cancer did not report results on the association. Furthermore, two studies examined the relationship between HCC and fumonisin B1 exposure and did not find any statistical significance between them [44]. Lastly, one study examined the risk of cervical cancer with regards to zearalenone exposure and did not find any results that implied a causal link between zearalenone in the blood and the risk of cervical cancer in the participants.

## 4. Discussion

In this systematic review, the aim was to synthesize the available evidence from epidemiological studies on the effects of mycotoxin-linked mutations on the risks of cancer. It also provides a summary of the data on the association between mycotoxin exposure and cancer risk in humans. This review found relatively few studies on the subject. The majority of the studies focused on aflatoxin exposure. The sixteen studies included focused on the links between mycotoxin exposure and (1) primary liver cancer risk, (2) breast cancer risk, (3) cervical cancer risk (4) colorectal cancer risks and (5) oesophageal cancer risk. Meta-analysis results found no significant association between aflatoxins and risk of primary liver cancer (OR 1.50, 95% CI: 0.91, 2.08).

### 4.1. Mycotoxin-Linked Mutations and Increased Primary Liver Cancer Risks

Most publications in this study examined the association between aflatoxins and liver cancer, and the relationship between aflatoxin-linked mutations and liver cancer risk was confirmed. The results are in line with the conclusions of the World Cancer Research Fund (WCRF) that there is a strong association between dietary aflatoxin and the risk of liver cancer [50]. Furthermore, a study by Baertschi et al. [51] on experimental animals also observed the carcinogenic effects of aflatoxin B1 (AFB1) and aflatoxin G1 (AFG1). It was also found that aflatoxin carcinogenicity is the result of a genotoxic mechanism of action which involves the generation of a genotoxic epoxide metabolite, which invariably forms DNA adducts and modifies the TP53 gene [52]. Hepatocellular carcinoma (HCC) is the third leading cause of cancer-related deaths in the world [53]. Aflatoxins, (AFB1, AFB2, AFG1, AFG2, and AFM1), have been classified as carcinogenic to humans [54,55], based on experimental data and epidemiological studies in human populations. The liver serves as a target organ for aflatoxins, and studies have shown liver damage in fish, poultry, and primates after ingesting aflatoxin B1. Historically, numerous epidemiological studies in Asia and Africa have shown a relationship between high aflatoxin exposure and increased incidence of HCC [56]. Although chronic hepatitis B virus infection is the major risk factor for HCC, other environmental exposures, including aflatoxins in particular, have also been suggested to increase the risk [57]. Moreover, studies have demonstrated a linear correlation between serum AFB1, dietary exposure, and the risk of developing HCC [58]. Aflatoxins are metabolized by liver enzymes, and they generate reactive epoxide species that are able to form a covalent bond with guanine (aflatoxin-N7-guanine-adduct) which causes mutations [59,60]. AflatoxinB1 is metabolized by cytochrome P450 (CYP450) to form an unstable and highly reactive aflatoxin-8,9-epoxide, which binds to DNA or proteins such as albumin [3,61,62,63]. Moreover, there is an indication that AFB1-induced mutagenicity is attributable to the direct genotoxic mode of action [54,64]. Hence, if these mutations occur in oncogenes or tumour suppressors, these may cause the growth and spread of abnormal cells and result in cancer. Historically, aflatoxins are implicated with the incidence of hepatocellular carcinoma in low- and middle-income countries. This was suspected to be through the ingestion of home-grown agricultural crops [65,66]. However, one reviewed article reported no link between PLC and aflatoxin intake, hepatitis B infection, and certain dietary patterns [36]. The article was focusing on cholangiocarcinoma, a type of liver cancer, whereas most links between aflatoxins and liver cancer is commonly studied from the angle of HCC [50]. This suggests that various causations for liver cancer types explain the heterogeneity amongst reports [50]. In addition, due to the fact that different studies used different confounders, this can critically influence human epidemiological study results [50]. Nevertheless, since individual results from this systematic review ranged from very little association to a strong association, when the results were pooled in a meta-analysis, the overall meta-analysis result showed no significant association (OR 1.50, 95% CI: 0.91, 2.08) between aflatoxin exposure and primary liver cancer (Figure 2).

Additionally, no significant association was found in the two included studies investigating exposure to fumonisin B1 and HCC. However, consistent with IARC classifications, fumonisin B1 can possibly modify protein synthesis. Moreover, the obstruction of DNA synthesis can occur by higher concentrations in intestinal cells in vitro [67,68,69]. Another study has further demonstrated the use of urinary fumonisin B1 as an exposure matrix to assess exposure to fumonisin B1. This enhanced assessment could help future studies to ascertain associations between fumonisin B1 exposure and cancer risks [70,71]. Furthermore, in addition to the familiar matrices, two of the studies from China examined toenails as an exposure matrix [44]. While toenails are not known as reliable matrices for the assessment of fumonisin B1, neither has another study examined the half-life of fumonisin B1 in nails. Another study using laboratory animals showed that the levels of fumonisin B1 might be detected in hair after exposure [72], and may be of use in assessing human exposure to fumonisin B1 [44,73]. Other human studies thus far studying the association between fumonisin B1 and risk of liver cancer include [44,74,75,76,77].

### 4.2. Mycotoxin-Linked Mutations and Increased Breast Cancer Risks

Two case–control studies (incorporated into this review) conducted in Africa studied the links between zearalenone and its metabolites, e.g., α-zearalenol in relation to the risk of breast cancer. Although the results were conflicting, it is of note that different biological matrices were used, which cannot easily be compared. One of the studies examined urine [46] while the other used plasma [41]. The results suggested that α-zearalenol could play a possible role in triggering breast cancer [46]. Moreover, since zearalenone is similar in structure with oestrogen, it can exert an affinity for oestrogen receptors and have an adverse effect on the fertility of humans and farm animals [3]. Other studies also suggest that zearalenone and the metabolites could initiate cancer of the reproductive system in humans and animals [3,41].

Zearalenone was shown to be able to initiate cancer in rats, e.g., hepatocellular adenomas and pituitary tumours [78,79]. Conversely, mycotoxin biomarkers in plasma could not specify a causal association between exposure to zearalenone risk of breast cancer in one South African study [41]. However, further epidemiological studies are necessary to validate the likelihood of zearalenone to cause cancer in humans [79].

### 4.3. Mycotoxin-Linked Mutations and Increased Cervical Cancer Risks

An epidemiological study by Pillay et al., [41], in South Africa studied the relationship between zearalenone and cervical cancer, but no association was found [41]. Therefore, it could still be hypothesized that zearalenone could cause cancer of the genitals in humans, owing that it exerts oestrogenic activities in various species of animals. It was also shown to form DNA adducts in the genitals of rats, other rodents, and domestic animals, e.g., horses [41]. Hence, more research is needed to understand the association between zearalenone and the risk of cervical cancer in humans.

### 4.4. Mycotoxin-Linked Mutations and Increased Colorectal Cancer Risks

A study by Ouhibi et al. [47], in Tunisia studied the relationship between citrinine and patulin and colorectal cancer, but no association was found [47]. Therefore, it could still be hypothesized that CIT affects the kidney function in different species and degenerates the processes of the renal tubules [80] and patulin could cause mutagenic, immunotoxic, and genotoxic effects, with possible implications on the GIT tracts of rodents [81,82].

### 4.5. Mycotoxin-Linked Mutations and Increased Esophageal Cancer Risks

The authors found the co-occurrence of the mycotoxins (NEO, HT-2, and T-2) in the urine samples. While T-2 toxin was only present in the oesophageal cancer group, HT-2 was the most common mycotoxin in combination present in all the co-occurring samples, followed by NEO, which was present in two of the three multi-contaminated urine samples [48]. Although the relationship between mycotoxin exposure and oesophageal cancer incidence was not established, and since cancer is a multifactorial disease, other cofounding factors could play a role in the development of the disease.

## 5. Way Forward and Future Research

Mycotoxins are ubiquitously found all over the world, in many foods and feedstuffs, which may be chronically consumed by majority of people all around the world. Most studies investigating the associations between mycotoxin exposures and cancer risk have mainly concentrated on aflatoxins, while other important mycotoxins, for example, ochratoxins, etc., are not well dealt with. Therefore, having known mycotoxins to have toxigenic potentials, there is need for continuous research in order to understand the mechanisms of their carcinogenic effects, also taking into consideration the co-occurrence of different mycotoxins in food and the synergistic effects with other mycotoxins, for public health purposes and the prevention of economic losses.

### 5.1. Conclusions

This work has successfully assessed the link between mycotoxin-linked mutations and the risk of cancer. Mycotoxins are a ubiquitous contaminant of many foodstuffs and agricultural products, and the carcinogenic potency of some mycotoxins such as aflatoxins are already known. Thus, inasmuch as the meta-analysis result did not find a significant association, some experimental studies did establish the link between mycotoxin exposures and cancer risk. Additionally, many emerging mycotoxins have not been investigated with respect to their health outcomes. We therefore propose the need for these links to be confirmed and validated using more human epidemiological studies, taking into consideration mycotoxin co-occurrence in food and their synergistic potentials with one another. This will help guide public health policies and interventions.

### 5.2. Limitations of the Systematic Review

While we did a comprehensive search in the major electronic databases, it is possible that we could have missed some non-English studies. Furthermore, the comparability of the study results was limited because of the different study populations and different biological matrices used for the exposure assessments.

## Figures and Tables

**Table 1:** Characteristics of studies on association between mycotoxins linked mutations and cancer risks.

Ref.	First Author	Year	Study Population	Study Design	Follow-Up Duration	Exposure	Matrix
[35]	Bulatao-Jayme	1982	Philippines	Case control	N/R	Dietary	Urine
[36]	Parkin	1991	Thailand	Case control	1 year	Dietary	Blood and faeces
[37]	Chao	1994	Singapore	Case control	2 years	N/R	Blood and liver
[38]	Wang	1996	Taiwan	Case control	4 years 5 months	Environmental	Blood and urine
[39]	Omer	1998	Sudan	Case control	7 months	N/R	Food
[40]	Zang	1998	China	Case control	1 year 10 months	Dietary	Food
[41]	Pillay	2002	South Africa	Case control	N/R	N/R	Plasma
[42]	Wu	2008	Taiwan	Case control	10 years 5 months	Environmental	Urine
[43]	Wu	2009	Taiwan	Case control	13 years 5 months	Environmental	Urine
[44]a	Persson	2012	China	Case control	7 years 8 months	N/R	Toenails
[44]b	Persson	2012	China	Case control	10 years	N/R	Toenails
[49]	Chen	2013	China	Cohort	30 years	Dietary	Serum
[45]	Lai	2014	China	Case control	6 months	Environmental	Dust and serum
[46]	Belhassen	2015	Tunisia	Case control	6 months	N/R	Urine
[47]	Ouhibi	2020	Tunisia	Case control	N/R	N/R	Blood and urine
[48]	Niknejad	2021	Iran	Case control	N/R	N/R	Urine

**Table 2:** Newcastle–Ottawa Scale quality assessment of each included study.

Study	Selection	Comparability	Exposure/Outcome	Total Score
Bulatao-Jayme et al., 1982 [35]	***	*	**	6
Parkin et al., 1991 [36]	***	**	*	6
Chao et al., 1994 [37]	*	-	-	1
Wang et al., 1996 [38]	***	**	*	6
Omer et al., 1998 [39]	***	**	*	6
Zang et al., 1998 [40]	***	**	*	6
Pillay et al., 2002 [41]	*	*	-	2
Wu et al., 2008 [42]	****	**	*	7
Wu et al., 2008 [43]	****	**	*	7
Persson et al., 2012 [44]a	****	**	*	7
Persson et al., 2012 [44]b	****	**	*	7
Chen et al., 2013 [49]	-	*	-	1
Lai et al., 2014 [45]	****	**	*	7
Belhassen et al., 2015 [46]	**	*	***	6
Ouhibi et al., 2020 [47]	***	*	*	5
Niknejad et al., 2021 [48]	**	*	*	4

Stars (*, **, ***, ****) stand for one, two, three or four scores in the assessment of study quality using modified NOS.

**Table 3:** Summary of findings on association between mycotoxin-linked mutations and cancer risks.

Ref.	Author	Year	Sample Size	Mycotoxin	Technique	LOD:LOQ	Cancer Type (s)	RRs	ORs	95% CI
[35]	Bulatao-Jayme	1982	180	Aflatoxins	N/R	N/R	PLC	1/3.9/17.5/35.0	N/R	N/R
[36]	Parkin	1991	206	Aflatoxins	ELISA	N/R	PLC	N/R	1.4	0.8–14.6
[37]	Chao	1994	481	Aflatoxins		N/R	PLC:HCC	N/R	N/R	N/R
[38]	Wang	1996	276	Aflatoxins	ELISA	0.1 fm/ug	PLC:HCC	N/R	7.22	1.5–34.3
[39]	Omer	1998	58	Aflatoxins	HPLC	N/R	PLC:HCC	N/R	7.5	1.4–40.2
[40]	Zang	1998	267	Aflatoxins		N/R	PLC:HCC	N/R	16.44	1.67–61.65
[41]	Pillay	2002	106	Zearalenone α-zearalanol b-zearalenol	HPLC GC-MS	25 ng/mL	Breast: cervix	N/R	N/R	N/R
[42]	Wu	2008	364	Aflatoxin B1	ELISA	0.2 ng/mL	PLC:HCC	N/R	7.5	3.14–16.46
[43]	Wu	2009	1102	Aflatoxin B1	ELISA	N/R	PLC:HCC	N/R	5.5	1.3–23.4
[44]a	Persson	2012	551	FumonisinB1	HPLC-MS-MS	6 pg/L:20 pg/L	PLC:HCC	N/R	1.1	0.64–1.89
[44]b	Persson	2012	219	FumonisinB1	HPLC-MS- MS	6 pg/L:20 pg/L	PLC:HCC	N/R	1.47	0.70–3.07
[49]	Chen	2013	652	Aflatoxins	N/R	N/R	PLC	7.3, 3.4, 59.4	N/R	N/R
[45]	Lai	2014	218	Aflatoxins	ELISA	N/R	PLC:HCC	N/R	5.24	2.77–9.88
[46]	Belhassen	2015	110	α-zearalanol	UHPLC-MS/MS	0.2 ng/mL:0.7 ng/mL	Breast	N/R	1.54	1.10–2.77
[47]	Ouhibi	2020	100	Citrinin and Patulin	LC-MS/MS	1 ng/mL:2.88 ng/mL	Colorectal	N/R	N/R	N/R
[48]	Niknejad	2021	27	NEO, HT-2, T-2	GC-MS/MS	0.25:0.5 ug/L 1:2 ug/L 0.5:1 ug/L	Esophageal	N/R	N/R	N/R

Abbreviations: HCC, hepatocellular carcinoma; HBV, hepatitis B virus; LOQ, limit of quantification; LOD, limit of detection; N/R, not reported; OR, odds ratio; RR, relative risk; CI, confidence interval; PLC, primary liver cancer; 7.3,3.4,59.4 RR: 7.3 (men with HBV, no aflatoxin) RR: 3.4 (men with aflatoxin, no HBV) RR: 59.4 (men with HBV, urinary aflatoxin biomarkers); RR = 1; Light Aflatoxin, Heavy Alcohol, RR = 3.9; Heavy Aflatoxin, Light Alcohol, RR = 17.5; Heavy Aflatoxin, Heavy Alcohol, RR = 35.0.

## Data Availability

Not applicable.
